# Dramatic intracranial response to tepotinib in a patient with lung adenocarcinoma harboring *MET* exon 14 skipping mutation

**DOI:** 10.1111/1759-7714.13871

**Published:** 2021-02-03

**Authors:** Shinkichi Takamori, Taichi Matsubara, Takatoshi Fujishita, Kensaku Ito, Ryo Toyozawa, Takashi Seto, Masafumi Yamaguchi, Tatsuro Okamoto

**Affiliations:** ^1^ Department of Thoracic Oncology National Hospital Organization Kyushu Cancer Center Fukuoka Japan

**Keywords:** brain astasis, MET, met, non‐small cell lung cancer, tepotinib

## Abstract

Mesenchymal‐epithelial transition (MET) pathway activation is associated with the mechanisms that influence properties affecting cancer cell survival and invasiveness. The MET exon 14 skipping mutation (METex14del) is found in 2%–3% of patients with non‐small cell lung cancer (NSCLC). Previous studies reported that NSCLC patients harboring a METex14del responded well to MET‐tyrosine kinase inhibitors (TKIs), including tepotinib. Tepotinib is a highly selective, once‐daily oral MET inhibitor that has shown promising clinical activity in patients with NSCLC with METex14del. The Food and Drug Administration accepted a new drug application for tepotinib as a treatment for patients with metastatic NSCLC harboring METex14del in February 2021 [Correction added on 5 March 2021, after first online publication: the FDA approval date for tepotinib has been corrected from ‘September 2019’ to ‘February 2021’.]. However, in the previous clinical trials involving MET‐TKIs, only patients with stable central nervous system metastases were eligible, and those with untreated symptomatic brain metastases (BMs) were excluded. Therefore, the efficacy and safety of MET‐TKIs in that population remains unknown. We herein report a case of dramatic intracranial response to tepotinib in a patient with symptomatic BMs from lung adenocarcinoma harboring METex14del. In the current report, the symptoms derived from multiple BMs (headache and loss of appetite) rapidly disappeared, and brain magnetic resonance imaging (MRI) examination showed that all the lesions were too small to measure only 23 days after the commencement of tepotinib. For NSCLC patients with multiple BMs, whole‐brain irradiation is a standard‐of‐care therapy, but its adverse effects on neurocognition are concerning. Tepotinib might therefore be a therapeutic option for NSCLC patients with symptomatic multiple BMs harboring METex14del.

## Graphical abstract

In the previous clinical trials, patients with symptomatic brain metastases (BMs) from lung cancer harboring METex14del were excluded. We report a case of dramatic intracranial response to tepotinib in a patient with symptomatic BMs from lung adenocarcinoma harboring METex14del. Tepotinib might be a therapeutic option for lung cancer patients with symptomatic multiple BMs harboring METex14del.

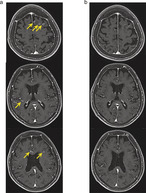

## INTRODUCTION

Lung cancer is one of the most devastating malignancies, and its prognosis remains poor.^1^ Non‐small cell lung cancer (NSCLC) accounts for 85%–90% of the disease, and advances in molecular‐targeted therapy have dramatically improved the prognosis of NSCLC patients,^2^, ^3^ Mesenchymal‐epithelial transition (*MET*) exon 14 skipping mutation (METex14del) is a splice‐site oncogenic mutation, which is shown in 2%–3% of NSCLC patients and is one of the therapeutic targets.^4^ MET pathway activation is associated with the mechanisms that influence properties affecting cancer cell survival and invasiveness.^5^ According to previous clinical trials, NSCLC patients harboring METex14del responded well to MET‐tyrosine kinase inhibitors (TKIs), including crizotinib, tepotinib, capmatinib, and cabozantinib.^6^, ^7^, ^8^, ^9^

Tepotinib is a highly selective, once‐daily oral MET inhibitor that has shown promising clinical activity in patients with NSCLC with METex14del.^6^ The Food and Drug Administration accepted a new drug application for tepotinib as a treatment for patients with metastatic NSCLC harboring METex14del in February 2021 [Correction added on 5 March 2021, after first online publication: the FDA approval date for tepotinib has been corrected from ‘September 2019’ to ‘February 2021’.]. The response rate of tepotinib has been reported to be 46% (95% confidence interval [CI]: 36–57), with a median duration of response of 11.1 months.^6^ The common adverse events (all grade) of tepotinib include peripheral edema (63%), nausea (26%), diarrhea (22%), blood creatinine increase (18%), and hypoalbuminemia (16%).^6^ The Food and Drug Administration approved the ArcherMET test as a companion diagnostic to identify METex14del in patients' tissue and liquid biopsy samples to identify those eligible for tepotinib. However, in the previous clinical trial, patients with symptomatic brain metastases (BMs) from NSCLC harboring METex14del were excluded, and the intracranial response to tepotinib remains unclear.^6^ We herein report a case of dramatic intracranial response to tepotinib in a patient with lung adenocarcinoma harboring METex14del.

## CASE REPORT

**FIGURE 1:**
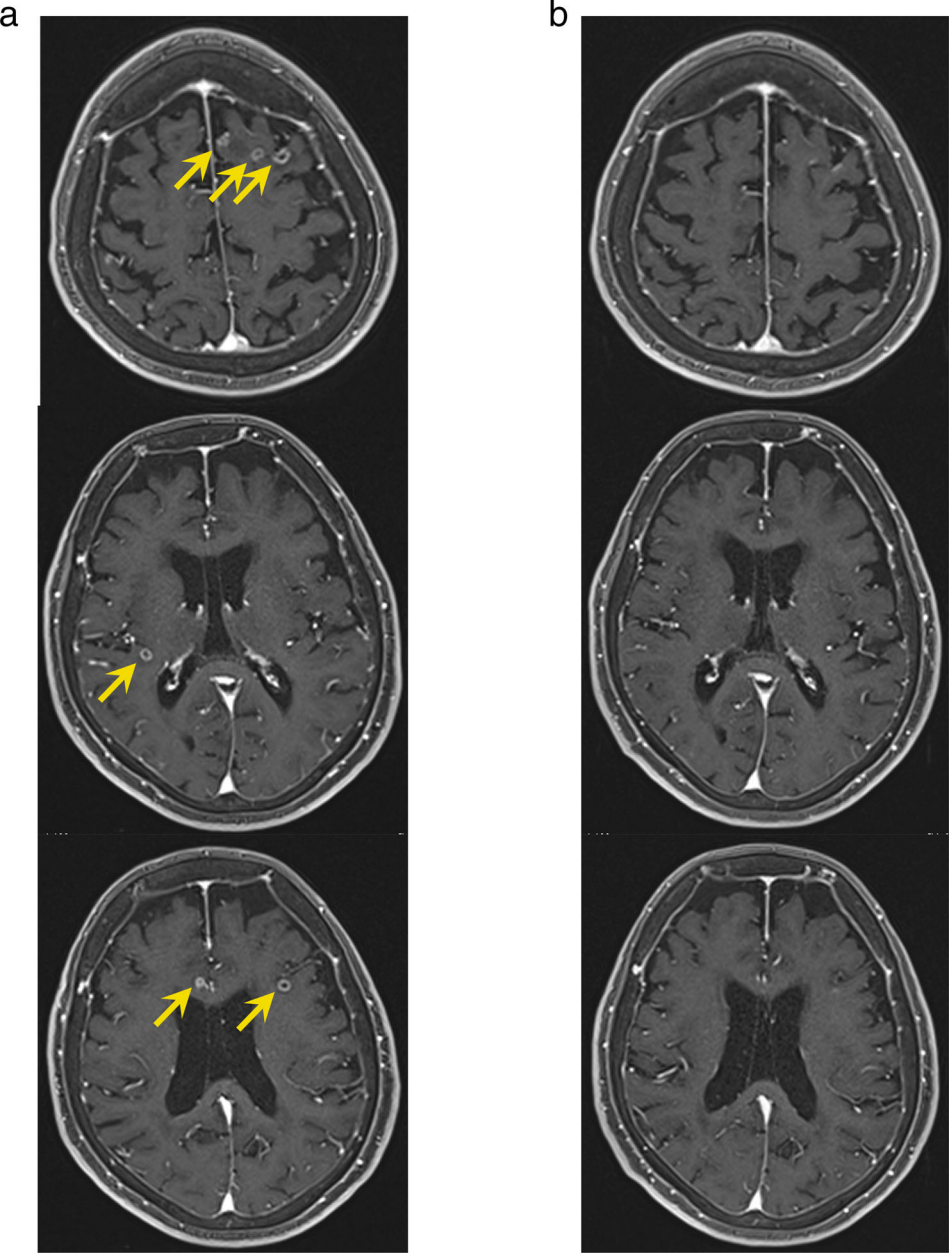
(a) Brain magnetic resonance imaging (MRI) showing multiple brain metastases (yellow arrows). (b) Twenty‐three days after commencing tepotinib, repeat brain magnetic resonance imaging showed that all the lesions were too small to measure

A 75‐year‐old woman was diagnosed with primary lung adenocarcinoma in the left upper lobe (cT1cN2M1a, cStage IVA). She underwent biopsy of the primary tumor, and METex14del was detected using next‐generation sequencing (NGS). The patient participated in a clinical trial, and received daily crizotinib (500 mg/day). Chest computed tomography (CT) showed a partial response after two months; however, after four months, the tumor developed resistance to crizotinib. As second, third, and fourth regimens, the patient received carboplatin (area under curve = 6) and pemetrexed (500 mg/m^2^), docetaxel (60 mg/m^2^), and nivolumab (240 mg/day), respectively. After developing resistance to nivolumab, she presented with headache and loss of appetite. Although chest and abdominal CT images revealed no progression, brain magnetic resonance imaging (MRI) showed multiple BMs (Figure 1(a)). A blood sample was submitted for liquid biopsy (Archer MET), and METex14del was negative. Because the original biopsied specimen was positive for METex14del with NGS, we administered tepotinib (500 mg/day) to the patient. After 10 days, her headache and loss of appetite improved rapidly. However, the patient presented with nausea 23 days after commencing tepotinib. With a suspicion of disease progression, we repeated the brain MRI examination, which showed that all the lesions were too small to measure (Figure 1(b)). The symptoms she experienced were considered adverse effects (AEs) of tepotinib. The patient has been attending our hospital and has received tepotinib for approximately two months without disease progression at the time of this report.

## DISCUSSION

Several studies have previously reported that NSCLC patients harboring METex14del respond well to MET‐TKIs.^6^, ^7^, ^8^ However, in most previous clinical trials involving MET‐TKIs in advanced NSCLC, only patients with stable central nervous system metastases were eligible, and those with untreated symptomatic BMs were excluded.^6^, ^7^, ^8^ Thus, this is the first report of a case of dramatic intracranial response to tepotinib in a patient with symptomatic multiple BMs from lung adenocarcinoma and harboring METex14del. Of note, the symptoms derived from multiple BMs rapidly disappeared, and radiological response was confirmed only 23 days after commencing tepotinib. For NSCLC patients with multiple BMs, whole‐brain irradiation (WBI) is a standard‐of‐care therapy, but its adverse effects on neurocognition are concerning.^10^ In patients with multiple BMs from epidermal growth factor receptor (EGFR)‐mutant NSCLC, EGFR‐TKIs were associated with significantly longer intracranial progression‐free survival (PFS) than with WBI.^10^ With regard to patients with multiple BMs from NSCLC with METex14del, tepotinib might also be a better first‐line therapeutic option, but this needs to be clarified in future clinical trials. It is also notable that intracranial response to tepotinib was observed after the patient developed resistance to crizotinib. Klempner et al.^9^ reported a similar case of intracranial activity of cabozantinib after intracranial progression following crizotinib therapy. Acquired mutations which impair the activity of crizotinib but can be overcome by tepotinib should be investigated in further studies.^11^

In conclusion, we herein report a case of dramatic intracranial response to tepotinib in a patient with lung adenocarcinoma harboring METex14del, and suggest that tepotinib might be a therapeutic option for NSCLC patients with symptomatic multiple BMs harboring METex14del.

## CONFLICT OF INTEREST

All authors declare no conflicts of interest in association with this report.
